# Obituary

**Published:** 1883-11

**Authors:** 


### Obituary.

Died in New Orleans, La., October 27, 1883, Dr. M. M. Oldham, of Springfield, Ohio.

For the last seven years Dr. Oldham has spent his winters in the South, with the hope of improving his health, which has been failing for several years.

He was born in Cambridge, Ohio, August 25, 1818.

He was educated at Granville, O., for the ministry and preached for some years. This he was compelled to abandon on account of throat troubles. After this he began the study of dentistry with Dr. J. Taft, his brother-in-law, at Xenia, O.

In 1854 he removed to Springfield, O., where he was very successful in the practice of his profession.

His first wife died in 1855, leaving in his charge five young children, all of whom survive him, and by their life and position reflect honor upon the memory of their departed father.

Dr. Oldham was married three times. In 1872 he married Miss E. Givin, of Steubenville, O., who survives him, and has the warmest love and affection of his children, not only for her intrinsic worth but especially for the faithfulness and devotion which she manifested to their father in his declining years. The afflicted family will have the sincere sympathy of all his friends and acquantances, of whom Dr. Oldham had a great many. He was eminently a Christian gentleman.

